# *In vitro* propagation of pascuita (*Euphorbia leucocephala* Lotsy): effects of different LED light colors on callus, shoots, and root induction

**DOI:** 10.5114/bta/220165

**Published:** 2026-06-27

**Authors:** Carlos de Jesus Morales-Becerril, Maria Teresa Beryl Colinas-Leon, Ramón Marcos Soto-Hernández, Ma Teresa Martínez-Damián, Natanael Magaña-Lira, O José Luis Rodríguez de la

**Affiliations:** 1 Universidad Autónoma Chapingo, Texcoco, Estado de México, Mexico; 2 Colegio de Postgraduados, Texcoco, Estado de México, Mexico

**Keywords:** artificial lighting, micropropagation, ornamental plants

## Abstract

**Background:**

The application of different light emitting diodes (LED) light colors in plant micropropagation is gaining prominence based on the morphological and physiological responses of plants when exposed to specific wavelengths and intensities of light. This study aimed to determine the effects of red, white, and blue LED light on the micropropagation of pascuita (*Euphorbia leucocephala* Lotsy), an ornamental plant native to Mexico and Central America.

**Materials and methods:**

Under *in vitro* conditions, the effects of the above mentioned three LED light colors at 90 µmol m^–2^/s^–1^ intensity were evaluated on callus formation, shoot induction, and plant growth. In the second phase, using the shoots developed in the previous stage, the influence of the three LED light colors on in vitro root induction was evaluated with or without indole-3-acetic acid (IAA) addition.

**Results:**

Red light enhanced callus induction and growth, with plant responses observed from the initial days of culture, while blue light favored shoot formation. During the rooting phase, red light demonstrated superior efficacy by inducing the highest number of roots, achieving a 100% rooting rate, regardless of IAA presence.

**Conclusions:**

The use of different LED light colors during pascuita micropropagation is an effective tool to modulate plant responses throughout the various stages of regeneration.

## Introduction

The success of *in vitro* plant propagation mainly depends on chemical and physical factors. Chemical factors include culture medium composition, supplementation with growth regulators, and addition of other growth-promoting compounds, while physical factors include temperature, humidity, and light conditions in the incubation rooms (Cavallaro et al. 2022). Regarding the illumination of the incubation rooms, the use of light emitting diodes (LED) light with different wavelengths during *in vitro* culture stages has emerged as a prevalent practice.

The potential of LED technology for *in vitro* plant propagation has been recognized since its initial development (Livadariu et al. 2023), which could be attributed to several favorable characteristics such as long operational lifespan, minimal heat emission, high energy efficiency, low power consumption, and capability to emit specific light wavelengths (Fan et al. 2022).

Among the various spectra of light, red and blue wavelengths exert prominent effects on plant morphology, influencing key processes such as cell differentiation, shoot elongation, root development, and leaf expansion (Kulus and WoŸny 2020; Lim et al. 2023; Ptak et al. 2024). Additionally, blue and red lights are critical for photosynthesis and functioning of the photosynthetic apparatus because these colors are primarily absorbed by chlorophylls (Nacheva et al. 2023). Plant exposure to these light wavelengths, particularly at elevated light intensities, can substantially modify the production of pigments and other secondary metabolites (Gupta and Sood 2023; Jiao et al. 2023; Livadariu et al. 2023). However, responses to these and other light wavelengths are dependent on the plant genotype (Fan et al. 2022; Livadariu et al. 2023), highlighting the need to investigate the response of each plant species under specific lighting conditions.

Pascuita (*Euphorbia leucocephala* Lotsy), an ornamental plant native to Mexico and Central America, is currently being explored for potential applications. Pascuita blooms during autumn and winter, and similar to the poinsettia (*Euphorbia pulcherrima*), it is marketed on a small scale in central and southern Mexico as a potted plant during the Christmas season (Colinas-León et al. 2024). Nevertheless, there are several challenges in the production of pascuita. This plant is typically propagated through cuttings; however, its low rooting rate has driven the search for more efficient asexual propagation methods. In this context, the manipulation of the light spectrum using LEDs was found to considerably improve adventitious root formation and shoot growth in stem cuttings of this species (Morales-Becerril et al. 2025). Nevertheless, the need for even more efficient and mass asexual propagation methods has further prompted the advancement of *in vitro* culture protocols (Colinas-León et al. 2024).

Colinas-León et al. (2024) conducted experiments to establish *in vitro* cultures of pascuita under different LED light colors. The authors reported that pascuita can be successfully established *in vitro* and that callus and shoot formation could be effectively induced through the application of different combinations of indole-3-acetic acid (IAA) and benzyl adenine (BA) at varying concentrations, together with different LED light colors at 10 µmol m^–2^/s^–1^ intensity, with red light showing particularly positive effects. Nonetheless, several aspects warrant further investigations, such as the interaction between LED light colors and plant growth regulators, impact of higher light intensities, and influence of light quality on rooting and plantlet acclimatization.

Given this background, this study aimed to evaluate the effects of white, red, and blue LED lights at 90 µmol m^–2^/s^–1^ intensity on callus, shoot, and root induction in pascuita under *in vitro* conditions. Two experiments were conducted: the first experiment evaluated the effects of these light colors on callus and shoot induction, while the second experiment analyzed root formation in the presence or absence of IAA. The results showed that blue light accelerates and enhances shoot induction, whereas red light promotes callus induction and growth. In the second stage, red light favored root induction and growth, regardless of IAA presence.

## Materials and methods

### Plant material and explant sterilization

The explants were obtained from 3-year-old *E. leucocephala* Lotsy mother plants, cultivated under greenhouse conditions in 11.6 l plastic pots with a substrate composed of peat, perlite, and compost in a 2 : 2 : 1 ratio. The explants (nodal fragments) were collected from the most recently formed whorl located below the apex in 3-month-old stems. After removing leaves and petioles, the explants were transported to the laboratory and submerged in an antioxidant solution containing 150 mg/l^–1^ citric acid and 100 mg/l^–1^ ascorbic acid.

The disinfection process involved washing the explants with soap, running water, and 5 drops of Tween^®^ for 10 min. The explants were then washed with a fungicide solution (Funlate^®^ 50; benomyl 50%) at 1 g/l^–1^ concentration and a bactericide (Intermicin^®^ 500; streptomycin, oxytetracycline, and tribasic copper sulfate) at 2 g/l^–1^ concentration for 10 min under constant agitation. The explants were then immersed in 10% commercial sodium hypochlorite for 5 min and finally rinsed with distilled water inside a laminar flow hood. Subsequently, the explants were kept in the abovementioned antioxidant solution.

### Light treatments

The experiments were conducted in light isolation chambers measuring 40 × 40 × 80 cm (length × width × height), lined with an aluminum foil. These chambers were placed in growth rooms maintained at 22°C ±2°C. For illuminating the chamber, white (TIANLAI TLRL-02, Tianlai, China), red, and blue (HYDROFARM PPB1004, Hydrofarm, USA) LED lamps were used. Their spectral characteristics are provided in Table 1 and were measured with an Apogee StelarNet PS-300 spectroradiometer (StellarNet, USA). The height at which the LED lamps were installed was adjusted to achieve an average light intensity of 90 µmol m^–^2/s^–1^ on the chamber floor (Apogee QMSW-SS, Apogee Instruments, USA). The photoperiod was set to 16 h.

**Table 1:** Spectral characteristics of the light treatments used in this study

Light color	Peak emission (nm)	Photon flux density (%)				
		UV (300–400 nm)	Blue (400–500 nm)	Green (500–600 nm)	Red (600–700 nm)	Far red (700–800 nm)
White	445–556	0.76	35.79	42.95	18.15	2.35
Red	660	0.17	0.29	0.85	96.97	1.71
Blue	459	0.43	97.78	1.55	0.11	0.13

UV – ultraviolet.

### Callus and shoot induction

The nodal segments were divided into four parts, and each fragment was placed in a test tube (Colinas-León et al. 2024) containing 20 ml of modified WPM (woody plant medium) with the following composition (Martínez-Villegas et al. 2015): NH_4_NO_3_, 400 mg/l^–1^; Ca(NO_3_)_2_ · 4H_2_O, 695 mg/l^–1^; MgSO_4_, 370 mg/l^–1^; KH_2_PO_4_, 170 mg/l^–1^; Na_2_EDTA · 2H_2_O, 37.2 mg/l^–1^; FeSO_4_ · 7H_2_O, 27.8 mg/l^–1^; H_3_BO_3_, 6.2 mg/l^–1^; MnSO_4_ · 4H_2_O, 22.3 mg/l^–1^; ZnSO_4_ · 7H_2_O, 8.6 mg/l^–1^; Na_2_MoO_4_ · 2H_2_O, 0.25 mg/l^–1^; and CuSO_4_ · 5H_2_O, 0.25 mg/l^–1^. The medium also contained 3% sucrose, 100 mg/l^–1^ myo-inositol (Merck), 0.4 mg/l^–1^ thiamine-HCl (Sigma Aldrich), 300 mg/l^–1^ PVP (polyvinylpyrrolidone, Sigma Aldrich), 5 µmol IAA (Sigma Aldrich), and 12 µmol BA (Sigma Aldrich). The pH of the medium was adjusted to 5.7, and 0.7% agar (Sigma Aldrich) was added. Thirty replicates were established for each treatment condition.

The cultures were maintained for 30 days. At 10-day intervals, callus or shoot appearance, the percentage of callus coverage on the explants (visual assessment), and the number and average length of shoots were measured. At the end of the culture period, 10 shoots were sampled from each treatment to quantify the number of leaves, single-photon avalanche diode (SPAD) units (Konica Minolta SPAD-502PLUS, Konica Minolta, Japan), and fresh weight (Mettler^®^ analytical balance Model AJ150L). Dry weight was determined by drying the samples in a forced-air oven (Aparatos Márquez^®^) at 60°C until constant weight was achieved.

### Root induction

For root induction, 20 shoots measuring 1 to 2 cm in length were subcultured for each light treatment. The same culture medium (without growth regulators) used for callus induction was employed in this stage. Half of the shoots were cultured in medium supplemented with 2.5 µmol IAA, and the remaining shoots were grown in medium without the auxin. The shoots subcultured for rooting were established under the same light treatment from which they originated.

The cultures were maintained for 40 days. At 10-day intervals, the percentage of explants with root induction and the number of roots and their length were recorded.

### Acclimatization and transfer of seedlings to the greenhouse

Forty days after the initiation of root induction, the seedlings were removed from the test tubes, and the roots were carefully washed with running tap water to eliminate any residual culture medium. Subsequently, the seedlings were transferred to plastic trays with a specialized dome to regulate air intake. The dome remained closed for the first three days, was opened to 50% during the subsequent week, and was fully opened during the last week. The cavities in the 50 ml tray were filled with a substrate formulation comprising peat, perlite, and vermiculite in a 3 : 1 : 1 ratio. The planted trays were then placed in a greenhouse environment (28°C ±5°C, 60% ±15% relative humidity) and kept under shaded conditions for 20 days. Finally, the seedlings were transferred to 0.78 l pots containing a substrate comprising peat, perlite, and compost in a 3 : 2 : 1 ratio for their further growth and development.

### Data analysis

The results for shoot number, shoot length, fresh weight, dry weight, number of leaves, and SPAD units were analyzed by one-way analysis of variance (ANOVA), followed by Duncan’s multiple range test. Data involving percentages and the number and length of roots were evaluated by Kruskal-Wallis test. All statistical analyses were performed using SAS 9.0 software (SAS Institute Inc., Cary, NC, USA). Statistical significance was considered at *p* ≤ 0.05.

## Results

### Callus and shoot induction

**Figure 1:**
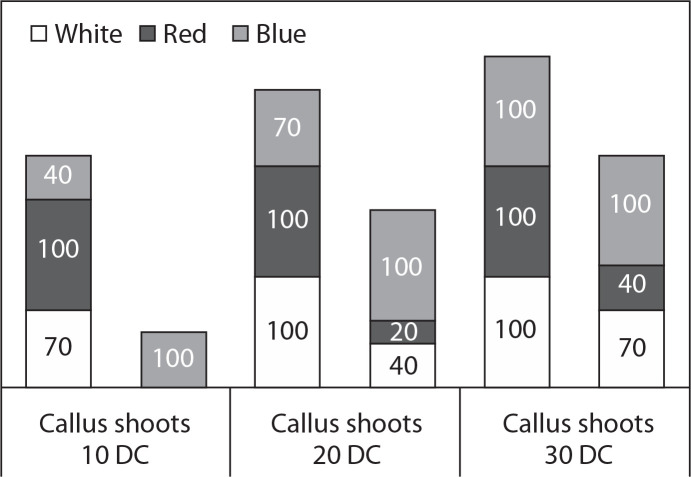
Effect of different light emitting diodes (LED) light colors on the percentage of explants that formed calli or shoots of *Euphorbia leucocephala* during three *in vitro* cultivation periods. DC – days of cultivation

The different light treatments induced varied responses in callus and shoot formation (Figure 1). Calli and shoots were successfully generated across all light treatments. Under red light, 100% of the explants formed calli within 10 days, while shoot formation was observed only under blue light in the same period.

**Figure 2:**
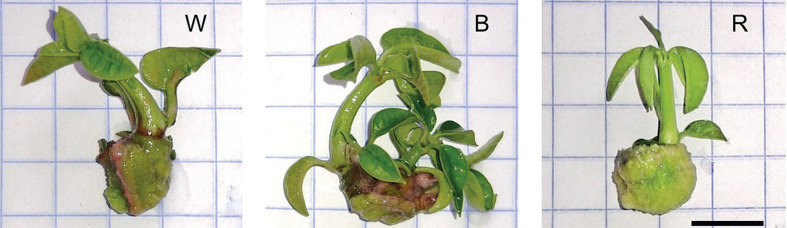
Effect of three light emitting diodes (LED) light colors on the multiplication and *in vitro* growth of *Euphorbia leucocephala* shoots after 30 days of cultivation. W – white LED, B – blue LED, R – red LED. Scale bar = 1 cm

Calli and shoots were simultaneously formed in the same explant under all treatments; however, the percentage of explants covered by callus was higher under red light, reaching 92% within the first 10 days of cultivation (Table 2). In contrast, the lowest callus coverage was recorded under blue light, with certain areas showing no signs of callus formation (Figure 2).

**Table 2:** Effect of different light emitting diodes (LED) light colors on the percentage of the explant covered with callus and the number and length of shoots of *Euphorbia leucocephala* formed during three *in vitro* cultivation periods

Days of cultivation	Parameter	White	Blue	Red
10	Callus coverage (%)	68.0^a^	18.0^b^	92.0^a^
	Shoot number	0.7^b^	2.3^a^	0.2^b^
	Shoot length (cm)	0.84^a^	0.93^a^	0.34^b^
20	Callus coverage (%)	77.0^a^	18.5^b^	92.0^a^
	Shoot number	0.8^b^	2.9^a^	0.3^a^
	Shoot length (cm)	1.87^a^	1.08^b^	0.66^b^
30	Callus coverage (%)	79.0^b^	41.0^c^	100.0^a^
	Shoot number	1.1^b^	3.0^a^	0.5^c^
	Shoot length (cm)	2.50^a^	1.60^b^	0.67^c^

Mean values followed by the same letter in the same row are not significantly different (Duncan *p* ≤ 0.05. Kruskal-Wallis *p* ≤ 0.05, for callus coverage).

The light treatments significantly affected shoot formation and growth (Table 2). On all three evaluation dates, blue light treatment yielded the highest number of shoots per explant, while red light treatment resulted in the lowest number of shoots. The shoots cultivated under white light exhibited the greatest length at both 20 and 30 days of cultivation, whereas those grown under red light had the shortest lengths on average (< 1 cm) on all evaluation dates.

Under blue light condition, a greater number of shoots were formed per explant, together with the highest recorded number of leaves per shoot (Table 3), thereby contributing to the maximum fresh and dry weight observed under this light treatment. Although the red light treatment resulted in the lowest fresh and dry weight, the differences were not statistically significant (*p* > 0.05) when compared with the values recorded under white light treatment.

**Table 3:** Effects of different light emitting diodes (LED) light colors on different variables evaluated in *Euphorbia leucocephala* shoots at 30 days of *in vitro* culture

LED color	FW	DW	Leaves per shoot	Chlorophyll (SPAD)
White	0.281^b^	0.028^b^	6.0^a^	29.10^a^
Blue	0.521^a^	0.055^a^	6.5^a^	27.27^a^
Red	0.304^b^	0.027^b^	4.5^b^	19.10^b^

Mean values followed by the same letter in the same row are not significantly different (Duncan *p* ≤ 0.05). FW – fresh weight, DW – dry weight, SPAD – single-photon avalanche diode.

SPAD units, which serve as an indicator of chlorophyll content by measuring the greenness of leaves, emerged as a significant variable in this study. Shoots exposed to white and blue light showed the highest SPAD values, while those grown under red light had the lowest SPAD values (Table 3).

### Root induction

**Figure 3:**
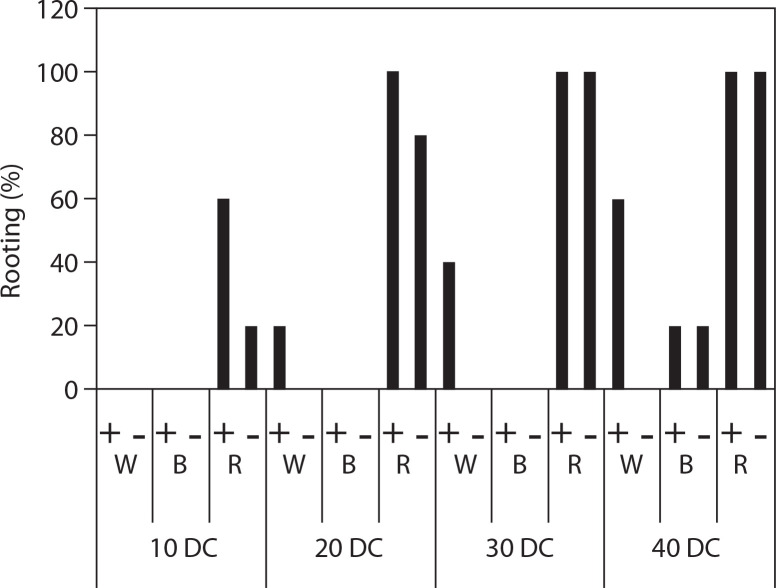
Effects of different light emitting diodes (LED) light colors and the presence of indole-3-acetic acid (IAA) on the rooting percentage at different days of *in vitro* culture. W – white, B – blue, R – red, “+” – with IAA, “–” – without IAA, DC – days of cultivation

The different LED light colors and the presence of IAA in the medium remarkably affected the rooting of shoots (Figure 3). Red light stimulated the rooting process, with root emergence observed as early as the first 10 days of cultivation (Figure 3). Under red light treatment, regardless of the presence or absence of IAA, 100% of the shoots exhibited root development within 30 days of cultivation. Furthermore, following white light treatment, roots were observed only in the presence of IAA, whereas under blue light, root development was recorded only after 40 days of cultivation, regardless of IAA presence/absence.

**Figure 4:**
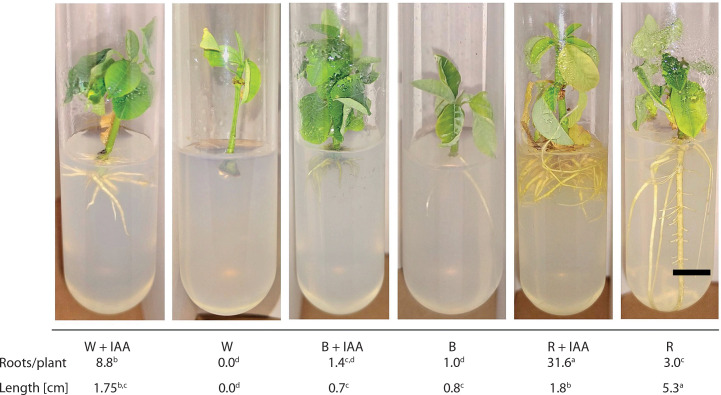
Effect of three light emitting diodes (LED) light colors and presence of indole-acetic acid (IAA) on roots formed in *Euphorbia leucocephala* shoots after 40 days of *in vitro* culture. W – white, B – blue, R – red. Scale bar = 1 cm. Mean values followed by the same letter in the same row are not significantly different (Kruskal-Wallis *p* ≤ 0.05)

Under red light condition, the cultivation period of 40 days yielded the highest number of roots (Figure 4), with an average of 31.6 and 3.0 roots per plantlet in the presence and absence of IAA in the medium, respectively. The lowest number of roots was recorded following blue light treatment, whereas no root formation was observed under white light treatment without IAA addition.

The light color and IAA presence in the culture medium also influenced root growth (Figure 4). The shoots rooted under red light in a medium without IAA showed the longest roots (5.3 cm), while the shortest roots were observed under blue light (0.7–0.8 cm).

### Acclimatization of seedlings and transfer to the greenhouse

**Figure 5:**
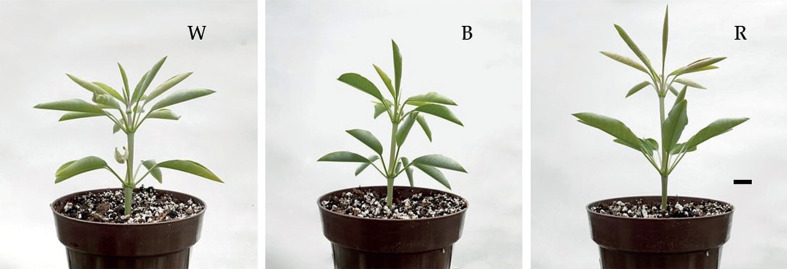
*Euphorbia leucocephala* plants from *in vitro* propagation under different light emitting diodes (LED) light colors plus indole-acetic acid (IAA), after 50 days of acclimatization in the greenhouse. W – white, B – blue, R – red. Scale bar = 1 cm

The plantlets were acclimatized successfully. All plantlets extracted from the test tubes exhibited effective adaptation to the greenhouse conditions. Notably, 50 days post-transfer, they appeared visibly healthy and showed adequate growth (Figure 5).

## Discussion

### Callus and shoot induction

Light serves as a critical environmental factor affecting plant physiology, growth, and development (Fan et al. 2022; Livadariu et al. 2023). Various studies have examined the effects of light conditions on the establishment and regeneration of different plant species under *in vitro* settings and concluded that alterations in light intensity, photoperiod, or light quality directly impact the physiological and morphological aspects of plant growth (Fan et al. 2022).

Previous research on *E. leucocephala* (Colinas-León et al. 2024) demonstrated that different LED light colors influence *in vitro* establishment, callus formation, and shoot generation, with red light showing the highest efficiency in promoting callogenesis and adventitious shoot formation. However, the present study revealed that variations in light intensity also alter the expected responses, highlighting the importance of this parameter in *in vitro* development.

*In vitro* propagation of plants involves multiple stages, with the initial phases focused on callus induction and proliferation as well as shoot formation. Our results indicate that red light enhances callus induction, with 100% of explants forming calli in the first 10 days of culture; in contrast, this percentage was reached at 20 and 30 days under white light and blue light conditions, respectively (Figure 1). Red light not only accelerated callus induction but also increased the proliferation rate, with explants showing a callus coverage percentage exceeding 90%; in contrast, these values were significantly lower following treatment with white or blue light (Table 2). Similar beneficial effects of red light on *in vitro* callus generation and proliferation have been documented in cotton (Yu et al. 2019) and *Withania somnífera* (Adil et al. 2019).

These findings can be explained by the differential impact of LED light colors on internal hormonal balance and the expression of genes associated with callus formation (Adil et al. 2019). The ideal auxin-to-cytokinin ratio for callus induction is species-specific and environmentally dependent (Sidik et al. 2024). Yu et al. (2019) demonstrated that red light enhanced endogenous IAA levels in cotton, resulting in a balanced ratio of approximately 0.5 between IAA and zeatin; conversely, exposure to blue light shifted the hormonal balance toward higher cytokinin concentrations, potentially explaining the low formation of callus in this light treatment.

Blue light emerged as the most effective treatment for shoot formation. Under blue light condition, new shoots were observed as early as 10 days after culture initiation, whereas no shoots were recorded under white or red light condition at the same time point (Figure 1). Additionally, blue light substantially enhanced shoot production, with 3.0 shoots per explant at 30 days, compared to 1.1 and 0.5 shoots under white and red light treatments, respectively (Table 2). This finding corroborates previous studies, wherein blue LED light was found to promote shoot proliferation in species such as *Stevia rebaudiana* (Ptak et al. 2024) and *Chinchona officinalis* (Vivanco-Galván et al. 2022) compared to red and white light. As noted in callogenesis, the observed effect may be associated with internal hormonal changes that create optimal conditions for shoot formation (Li et al. 2017; Yu et al. 2019).

In the present study, shoot proliferation primarily resulted from the sprouting of pre-existing meristems in the explants rather than from induced formation of new meristems (Figure 2). This finding contrasts with previous reports that indicate the ability of blue light to induce new meristems while suppressing their sprouting (Cavallaro et al. 2022; Geng et al. 2015). This divergence may be attributed to differences in genotype, explant type, and the phenological stage of the donor plant.

Shoots developed under white light exhibited the greatest elongation, producing the longest shoots under this condition (Table 2). Previous research has consistently indicated that red light is most effective in promoting shoot elongation, while blue light inhibits this process (Manivannan et al. 2021; Cavallaro et al. 2022; Fan et al. 2022; Lim et al. 2023; Ptak et al. 2024). However, in the present study, shoot induction occurred more slowly under red light than under white or blue light (Figure 1), which allowed greater shoot growth under white light over time.

Shoots cultured under blue light exhibited the highest values in both fresh and dry weight as well as the greatest number of leaves, although blue and white light treatments showed no significant difference in leaf number (Table 3). Comparable findings were reported by Li et al. (2017) in their study on grape, where they attributed these effects to blue light-induced overexpression of histone-related genes, which positively correlated with cell division. Different LED light colors also influence the internal hormonal balance of plants (Yang et al. 2015); notably, compared to red light, blue light exhibits greater efficacy in increasing photosynthetic rates (Guo et al. 2023), thereby directly influencing dry matter accumulation in *in vitro* plantlets.

Light plays a crucial role in chlorophyll biosynthesis. Different light colors can affect plant pigment production by activating photoreceptors (Cavallaro et al. 2022). In the present study, the highest chlorophyll concentrations (quantified in SPAD units) were recorded for plants exposed to white and blue light treatments (Table 3). These results align with multiple studies that identify blue light as a key promoter of chlorophyll biosynthesis (Li et al. 2017; Adil et al. 2019; Chen et al. 2019; Lim et al. 2023; Nacheva et al. 2023); this effect is attributed to blue light’s ability to regulate the over-expression of genes involved in chlorophyll synthesis pathways (Chen et al. 2019).

## Root induction

Rooting is a pivotal phase in *in vitro* plant propagation. The use of different LED light colors during this stage significantly influences the overall outcomes (Fan et al. 2022). The present study evaluated the effects of white, red, and blue LED light, with or without the inclusion of IAA in the culture medium, on root induction and growth. The results showed that red light enhanced root induction (Figure 4) and increased the number of roots formed (Figure 5), both in the presence and absence of IAA. These findings are consistent with previous research identifying red light as the most effective wavelength for root induction (Manivannan et al. 2021; Cavallaro et al. 2022; Fan et al. 2022; Gupta and Sood 2023; Nacheva et al. 2023). In contrast, some studies observed that, compared to red and white light, blue light is more effective in inducing adventitious rooting under *in vitro* conditions (Cavallaro et al. 2022; Fan et al. 2022; Guo et al. 2023), suggesting that these responses are genotype-dependent.

Our results revealed a synergistic interaction between different light colors and the presence of IAA in the culture medium (Figures 4 and 5); the addition of auxin further enhanced root induction and root number. Auxins play a key role in facilitating adventitious root development (Adem et al. 2024); moreover, seedling exposure to blue and red light promotes the over-expression of genes associated with auxin production, root formation, and root branching (Yun et al. 2023; Zeng et al. 2025). These observations suggest that adventitious roots can be induced under blue and red light and that this effect can be further enhanced by incorporating synthetic auxins in the culture medium.

Light quality provided by LEDs enhances seedling performance during the acclimatization process in greenhouse environments, particularly in plants with a well-established root system or when specific light wavelengths that promote rooting are utilized (Fan et al. 2022). In the present study, greenhouse acclimatization was successful for all rooted plantlets, with no significant differences observed among the treatments 50 days after transplanting to pots.

Based on the results of the present study, we suggest the following strategy to establish an effective *in vitro* propagation method for *E. leucocephala*: use of blue LED light during the initial phase to promote shoot formation from axillary meristems, followed by use of red LED light to promote root induction. This approach would shorten the time required to obtain plantlets compared to that needed following continuous white light exposure. Furthermore, we suggest the use of red LED light to improve the callus formation process. Future studies should prioritize the evaluation of this sequential lighting approach by conducting experiments that assess the effects of changing light colors at different stages on the *in vitro* propagation of *E. leucocephala*.

## Conclusions

The different LED light colors tested influenced various stages of *E. leucocephala* micropropagation. Red LED light efficiently promoted callus induction and proliferation, while blue LED light enhanced shoot formation and growth, resulting in the highest chlorophyll concentrations (SPAD units) and fresh and dry weight. The use of red LED light was the most effective approach for root induction, achieving 100% rooting success at 30 days, with the highest number and length of roots. Future research should focus on developing an optimized *in vitro* propagation protocol for *E. leucocephala*, utilizing blue LED light for shoot induction followed by red LED light for root formation; this approach could shorten propagation time compared to using white light alone.
